# T as a Biomarker for IDH1 Mutation Status in a Glioma Mouse Model

**DOI:** 10.1002/nbm.70214

**Published:** 2025-12-23

**Authors:** Hannah J. S. Ehler, Saki Sultana, Christa Davis, Kimberly Brewer, James Rioux

**Affiliations:** ^1^ Department of Physics & Atmospheric Science Dalhousie University Halifax Nova Scotia Canada; ^2^ School of Biomedical Engineering Dalhousie University Halifax Nova Scotia Canada; ^3^ IWK Health Centre Halifax Nova Scotia Canada; ^4^ Department of Diagnostic Radiology Dalhousie University Halifax Nova Scotia Canada; ^5^ Department of Microbiology & Immunology Dalhousie University Halifax Nova Scotia Canada; ^6^ Nova Scotia Health Halifax Nova Scotia Canada

**Keywords:** brain, glioma, IDH1, MRI, preclinical, quantitative imaging, T
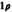

## Abstract

Glioma is a common and often aggressive malignant brain cancer for which treatment is in part dependent on the mutation status of the IDH gene. Current diagnostic methods require a biopsy and genetic analysis to obtain IDH status, which can have lengthy wait times. T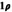, the spin–lattice relaxation in the rotating frame, has shown potential to serve as a faster, non‐invasive means of IDH1‐typing that could be implemented at clinically relevant field strengths; however, there have been few studies to date that have explored its utility. This study consisted of three groups of five mice: naïve controls, IDH1‐wild‐type glioma bearing and IDH1‐mutant glioma bearing, imaged once weekly with a T
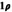
‐prepped EPI sequence. It was found that IDH1‐mutant gliomas exhibited significantly higher T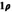 values in the tumour compared with the brain, while IDH1‐wild‐type gliomas presented similar T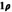 values in the tumour and brain. T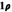 was found to be sensitive to whole‐brain changes linked to glioma and IDH status, although further investigations into the confounding effects related to T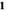 and T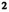 differences are needed. A measurement of tumour T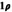 normalised to the brain (ΔT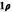) was able to distinguish between IDH1‐mutant and ‐wild‐type glioma, with IDH1‐mutant mice exhibiting an average ΔT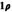 of ∼29% and IDH1‐wild‐type mice having an average ΔT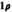 of only ∼3%. ΔT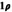 may thus have the capacity to serve as a non‐invasive biomarker for IDH1 typing.

## Graphical abstract

Mice bearing IDH1‐mutant and ‐wild‐type glioma underwent quantitative $T_{1\rho }$ imaging over the course of tumour development. It was found that while IDH1‐wild‐type tumours present similar $T_{1\rho }$ values as the brain, IDH1‐mutant tumours exhibit significantly higher $T_{1\rho }$ values compared with the brain. $T_{1\rho }$ may thus serve as a faster, non‐invasive biomarker of IDH1 status in glioma.

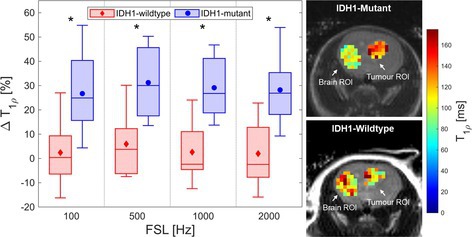

**Abbreviations**
2D: two dimensional
3D: three dimensional
CEST: chemical exchange saturation transfer
EPI: echo planar imaging
ESP: echo spacing
ETL: echo train length
FDR: false discovery rate
FOV: field of view
FSE: fast spin echo
FSL: spin‐lock amplitude
IDH: isocitrate dehydrogenase
MRF: magnetic resonance fingerprinting
MRI: magnetic resonance imaging
RF: radiofrequency
ROI: region of interest
TE: echo time
TR: repetition time
TSL: spin‐lock duration
WHO: World Health Organization

## 1 Introduction

Glioma is the most common cancer of the central nervous system, comprising 80% of malignant brain tumours [1]. The most recent classification system of glioma by the WHO (2021) categorises adult diffuse gliomas based on a combination of histological and molecular features, with one of the primary markers being isocitrate dehydrogenase (IDH) molecular status [2]. The IDH genes IDH1 and IDH2 are responsible for production of the enzymes IDH1 and IDH2, which play a key role in cell metabolism [3, 4]. These genes can have mutations which affect glioma growth and grading, with IDH1 mutations being more commonplace than IDH2 mutations [5, 6]. Gliomas which bear no mutation in the IDH1 or IDH2 gene (called ‘wild‐type’ gliomas) are classified as glioblastoma (Grade 4), while IDH‐mutant gliomas are classified as either astrocytoma (Grade 2–4) or oligodendroglioma (Grades 2 and 3) depending on other genetic/molecular markers (e.g., 1p/19q codeletion status) [2, 7]. IDH1‐mutant gliomas are typically less aggressive and more responsive to therapy than wild‐type gliomas, resulting in improved prognosis for patients [8, 9, 10]; meanwhile, wild‐type gliomas have a median overall survival time of only 12 months, with less than 5% of patients surviving to 5 years [11].

Determining IDH1 status of glioma is an important step in identifying tumour severity and will inform treatment course for the patient. Current diagnostic methods require a biopsy followed by genetic analysis of the tumour tissue [2], both of which can have lengthy wait times of up to a few weeks [12, 13, 14]. A reliable and non‐invasive IDH1 assessment tool could potentially reduce the time it takes to determine and implement a treatment plan for these patients.

Though quantitative magnetic resonance imaging (MRI) methods are being explored as faster, non‐invasive alternatives for IDH1 typing, they have yet to be implemented clinically [15]. Chemical exchange saturation transfer (CEST) [16], a technique which indirectly probes chemical content and concentration via chemical exchange between water and the molecular environment, has been shown to be effective in determining IDH1 status [15, 17, 18] and other genetic [19] and molecular [20] markers in glioma, as well as in glioma grading [21, 22, 23]. However, because CEST requires a separation of resonant frequencies between water and other metabolites [24, 25], this technique is generally only effective at high field strengths (≥3 T) [26], prohibiting its use in most clinics, which operate lower field scanners.

Quantitative T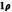 imaging is an alternative MRI technique which may help to bridge this gap in clinical utility. T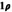 is the spin–lattice relaxation time in the rotating frame of reference [27]; it is characterised by application of a ‘spin‐lock pulse’ which keeps protons in‐phase while undergoing longitudinal magnetisation decay. This spin‐lock pulse, which is applied at some strength (FSL) for some duration (TSL), sensitises T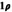 to lower frequency motional processes than conventional MR probes (kHz vs. MHz). These lower frequencies are associated with the chemical exchange between macromolecules (e.g., proteins) and free water [28, 29, 30, 31], causing T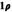 to be sensitive to conditions and changes at the cellular/molecular level, as well as in the tumour microenvironment [32, 33, 34]. Additionally, the strength of the spin‐lock pulse (FSL) may be adjusted to probe a specific frequency range and thus isolate exchange processes [35]; T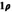 dispersion quantifies the change in T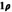 relaxation time between FSL [36]. Due to the nature of the spin‐lock pulse, T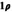 is largely independent of field strength [36] and is thus effective at low field [37, 38], making it ideal for clinical implementation and a potential alternative to CEST below 3 T [29].

Though there have been few studies on the application of T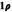 to glioma, they have shown T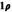 mapping in patients to be effective in assessing the histologic grade [39], IDH gene mutation status [39, 40, 41] and 1p/19q codeletion status [40], as well as in differentiating between oedema related to intracranial metastases and gliomas [42]. Preclinical studies have demonstrated T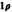 to be an early indicator of treatment response in rats with glioma [34, 43, 44] and have suggested different sensitivity to cellular and structural changes across FSL [44].

The goal of this study is to explore the potential for quantitative T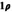 imaging to serve as a non‐invasive biomarker for IDH1 gene mutation status in glioma. In particular, we evaluate T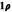 relaxation time and T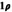 dispersion in a mouse model, comparing IDH1‐mutant and ‐wild‐type glioma‐bearing mice, as well as naïve controls.

## 2 Methods

### 2.1 Glioma Cells

U‐87 MG (HTB‐14) and IDH1 mutant‐U‐87 isogenic cell lines (HTB‐14IG) were obtained from American Type Culture Collection (ATCC, District of Columbia, USA) and cultured in Eagle's minimum essential medium (Corning Inc., Corning, NY, USA) supplemented with 1.5 g/L sodium bicarbonate, non‐essential amino acid, l‐glutamine, sodium pyruvate, 10% fetal bovine serum (heat‐inactivated serum; Corning Inc., Corning, NY, USA), 100‐U/mL penicillin and 100‐μg/mL streptomycin. Cell cultures were maintained at 37°C in a humidified atmosphere of 5% CO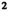 and harvested with TrypLE Express (Gibco, Thermo Fisher Scientific, Waltham, MA, USA) prior to implantation.

### 2.2 Animals

Experiments involving mice were carried out according to protocols approved by the University Committee on Laboratory Animals at Dalhousie University, Halifax, NS, Canada; mice were cared for following the standards of the Canadian Council on Animal Care.

**FIGURE 1:**
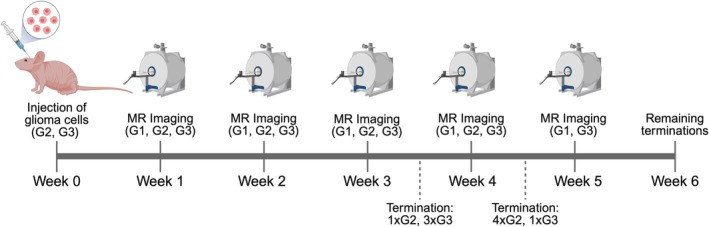
Timeline of the study. All mice from Group 1 (G1), Group 2 (G2) and Group 3 (G3) were imaged once weekly, beginning 1 week after glioma cell injections. Mice were terminated throughout the study if they became significantly ill or the tumour grew too large. Created in BioRender. Brewer, K. (2025) https://BioRender.com/n295w4l.

Mice were housed in sterile conditions at the IWK In Vivo Animal Care Facility and had ad libitum access to food and water. A total of 15 male NU‐$Foxn^{1nu}$ mice (6–8 weeks old, average body mass 28±2 g; Charles River Laboratories, Wilmington, MA, USA) were ordered together and randomly divided into three groups, with five mice per group. Group 1 was naïve controls, to account for possible age dependence of T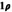 values [45, 46, 47], while Group 2 was implanted with glioma IDH1‐wild‐type cells and Group 3 implanted with glioma IDH1‐mutant cells. For Groups 2 and 3 mice, $5\times 10^{4}$ cells suspended in 2 μL of 1× Hank's Balanced Salt Solution (Corning Inc., Corning, NY, USA) were injected intracranially into the cerebral cortex just right of midline to promote tumour growth. Cell implantation was performed while mice were anesthetised, and analgesia was administered following the procedure. Tumour cell implantation marked Week 0 of the study; study timeline is displayed in Figure 1.

Each mouse was imaged once weekly (7±1 days apart), beginning 7 days post tumour cell implantation. During imaging, mice were anaesthetised with a 2.25% isoflurane/oxygen mixture. Respiration was monitored via a respiratory gating system (SA Instruments, Stony Brook, NY, USA) and isoflurane concentration was adjusted as needed to keep the mouse stable. Body temperature was monitored with a rectal probe and maintained constant at ∼37°C using a warm air heating system (SA Instruments, Stony Brook, NY, USA).

Mice were closely monitored throughout the study for changes in behaviour and health and were terminated if they became significantly ill or the tumour grew too large; all remaining mice were terminated on Week 6 of the study.

### 2.3 MRI

All imaging was conducted on a preclinical 3‐T scanner (Agilent, Santa Clara, CA, USA) with a quadrature RF coil.

**FIGURE 2:**
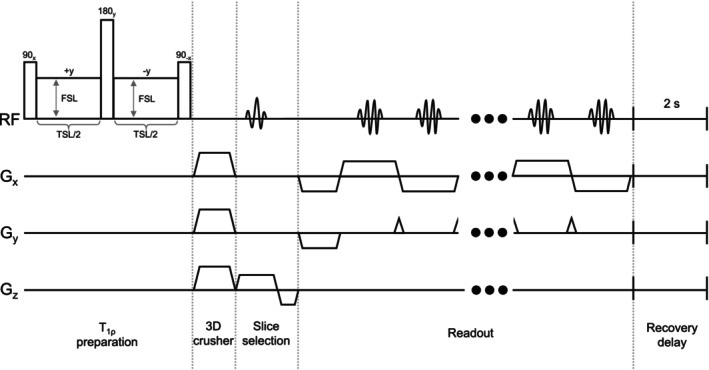
Pulse sequence diagram of the multislice T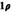 sequence created for this study, which consists of a composite spin‐lock module to prepare T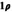 contrast followed by an EPI readout module and a 2‐s recovery delay to restore magnetisation (not to scale).

A 2D T
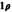
‐prepped imaging sequence was created using a composite spin‐lock module [48] followed by a 3D crusher gradient and a two‐shot centric EPI readout module [49], as depicted in Figure 2. Spin‐lock durations of TSL = [1, 15, 30, 45, 60, 75, 90, 105] ms were sampled to perform T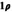 mapping, at spin‐lock frequencies of FSL = [100, 500, 1000, 2000] Hz to explore T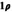 dispersion effects. Other parameters for the T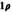 sequence were as follows: slices = 10 axial and interleaved (centred on brain); slice thickness = 1 mm; slice gap = 0.2 mm; FOV = 30×30 mm (due to EPI sequence limitations); acquisition matrix = 64×64; recovery delay = 2 s per slice; TE = 4.59 ms; duration = 3 min 29 s (to acquire all FSL for a given TSL).

In addition to T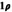 imaging, a T
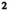
‐weighted fast spin echo (FSE) [50] anatomical image was acquired to permit brain and tumour delineation, adopting identical slice placement/positioning as the T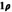 sequence. Parameters for the T
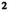
‐FSE sequence were as follows: slices = 10 axial and interleaved; slice thickness = 1 mm; slice gap = 0.2 mm; FOV = 17×17 mm; acquisition matrix = 64×64; TR = 1155.34 ms; TE = 56 ms; ETL = 16; ESP = 7; averages = 125; dummy scans = 2; duration = 9 min 39 s.

Due to the multislice nature of the T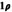 sequence, a correction was necessary to account for T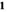 oversaturation effects in T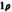 quantification (more in Section 2.4) [51]. Because this correction requires estimates of T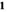 and T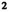, we acquired T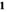 and T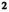 maps for a limited sample of mice from each group (five mice total) near the end of the study using a spiral magnetic resonance fingerprinting (MRF) sequence [52]. MRF images were positioned identically to the T
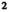
‐FSE and T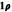 images and were acquired with the following parameters: slices = 20 linear; slice thickness = 1 mm; FOV = 20×20 mm; acquisition matrix = 64×64; duration = 28 min. Additional parameters and sequence information are given by Marriott et al. [52].

### 2.4 Image Analysis

All analysis was performed in MATLAB (R2024a; The MathWorks Inc., Natick, MA, USA) with the exception of region of interest (ROI) drawing which was done with the 3D Slicer software [53] via manual segmentation. The T
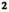
‐FSE and T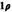 images were cropped to the same FOV (15.94×15.94 mm) to permit transferring ROIs between them, resulting in matrix sizes of 60×60 and 34×34 for the T
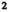
‐FSE and T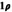 images, respectively. ROIs were drawn on the T
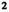
‐FSE anatomical images; delineations were made of the whole tumour (if applicable) and a region of healthy contralateral brain tissue. The average size and standard deviation of the brain ROIs were 18±2 mm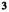 while those of tumours were 13±10 mm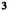.

Because the T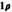 acquisition was multislice and the recovery delay (2 s) on the order of T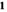 relaxation time, we applied the correction derived by Wheaton et al. [51] to account for residual T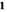 saturation affecting the longitudinal magnetisation. Assuming steady state is reached, the correction factor becomes

(1) $$\frac{M_{\text{z}}}{M_{0}}=M_{\text{sat}}\cdot \frac{1-e^{-\tau /T_{1}}}{1-M_{\text{sat}}e^{-\tau /T_{1}}}$$

where

(2) $$M_{\text{sat}}=e^{-\text{TSL}/T_{2\rho }}$$

and

(3) $$\frac{1}{T_{2\rho }}=\frac{1}{2}\left(\frac{1}{T_{1}}+\frac{1}{T_{2}}\right)$$

To perform the above correction, we used the average T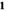 and T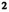 values of the brain and tumour for each group. These were obtained by registering the T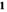 and T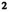 maps to the T
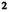
‐FSE images and extracting the average T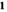 and T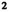 values in the brain and tumour ROIs for the five mice that this imaging was performed on (using 3D Slicer). These values were then averaged between mice of the same group (if applicable) to obtain group average T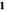 and T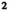 values for brain and tumour, which are listed in Table 1. These, along with TSL and τ=2000 ms (delay per slice), were input into Equations ((1), (2), (3)) to obtain the correction factor $M_{\text{z}}/M_{0}$, which the T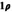 signal at each TSL was normalised by prior to T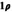 mapping.

**TABLE 1:** Average T and T values of brain and tumour ROIs for each group.

	T 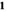 (ms)		T 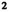 (ms)	
	Brain	Tumour	Brain	Tumour
Group 1	669	—	79	—
Group 2	670	827	98	120
Group 3	882	988	120	139

After correcting the T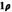 signal, maps of T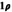 relaxation time were created for each FSL by fitting a monoexponential to each voxel:

(4) $$S(\text{TSL})=S_{0}\cdot e^{-\text{TSL}/{\text{T}}_{1\rho }}$$

where S(TSL) is the signal intensity at a given TSL, and $S_{0}$ and T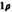 are free parameters. Mean T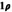 values of the brain and tumour ROIs were extracted from the T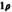 maps.

To probe the sensitivity of T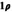 relaxation time to IDH1 mutation status while minimising the impact of individual variation in T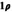 values, the normalised difference between mean tumour and brain T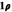 values (henceforth referred to as ‘ΔT
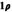
’) was calculated as the per cent difference between them:

(5) $$\Delta {\text{T}}_{1\rho }=\frac{{\text{T}}_{1\rho }(\text{tumour})-{\text{T}}_{1\rho }(\text{brain})}{{\text{T}}_{1\rho }(\text{brain})}\times 100\%$$

This was computed for each tumour‐bearing mouse (at all weeks in which they bore a tumour), for each FSL.

T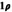 dispersion in the tumour ROIs for Groups 2 and 3 was quantified via the relative ratio between T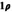 values measured at different FSL. Following the work by Kettunen et al. [43], this was calculated as

(6) $${\text{T}}_{1\rho }\;\text{Ratio}=\frac{T_{1\rho }({\text{FSL}}_{\text{high}})-T_{1\rho }({\text{FSL}}_{\text{low}})}{T_{1\rho }({\text{FSL}}_{\text{low}})}$$

for each combination of acquired FSL (100, 500, 1000, 2000 Hz), resulting in six T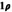 ratios computed for each tumour ROI.

### 2.5 Statistical Analysis

Age dependence of the T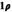 relaxation time was explored with Group 1. For each mouse, the average T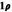 value of the brain ROI was plotted as a function of study week, for each FSL. Using the fitlm function in MATLAB, a linear regression was performed along with a t test to determine the slope of the fit and evaluate whether it was statistically different from zero. To account for multiple comparisons (four FSLs), the Benjamini–Hochberg correction [54] was applied with a false discovery rate of FDR = 5%. As no significant relationship was found between T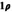 relaxation time and study week for any Group 1 mouse at any FSL, no age‐dependent corrections were performed.

To explore whether there existed a time dependence to T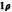 values over the course of tumour development, tumour T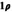 values were compared between each week at which tumours were present (Weeks 1–4). These were assessed for Groups 2 and 3 independently at each FSL via the Mann–Whitney *U* test [55] with the Benjamini–Hochberg correction (FDR = 5%). The same comparisons were made for brain T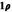 values, for each of the three groups. As no significant differences in tumour or brain T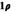 values were found between any weeks at any FSL for either group, T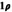 measurements were combined across weeks. This was done for data from Weeks 2 to 4, as at Week 1 only one mouse bore a tumour (which had an extremely small ROI, 2 mm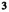), and no tumour‐bearing mice survived past Week 4. Tumour T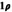 measurements from Weeks 2 to 4 were merged week‐wise at each FSL for each group. The same was done for brain T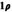 measurements, resulting in the total number of tumour/brain measurements in each group to be Group 1 = 0/15; Group 2 = 9/14; Group 3 = 7/12. All subsequent analyses used these combined samples.

T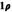 values were compared between groups and ROIs at each FSL using the Mann–Whitney *U* test with the Benjamini–Hochberg correction (FDR = 5%). To determine whether T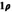 values could distinguish between healthy brain tissue and glioma, brain and tumour T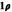 values were compared for Groups 2 and 3, independently. To explore whether T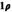 values could distinguish between IDH1‐mutant and ‐wild‐type glioma, tumour T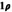 values were compared between Groups 2 and 3. Finally, the effects of IDH1‐mutant and ‐wild‐type glioma on healthy brain tissue were investigated by comparing T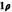 values from the healthy brain ROIs between all three groups.

The capacity for ΔT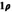 to distinguish between IDH1‐mutant and ‐wild‐type glioma was assessed by comparing ΔT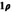 values between Groups 2 and 3 at each FSL using the Mann–Whitney *U* test with the Benjamini–Hochberg correction (FDR = 5%).

To explore the sensitivity of T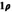 dispersion metrics to IDH1‐mutation status, the relative T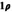 ratios of the tumour ROIs were compared between Groups 2 and 3 at each FSL combination using the Mann–Whitney *U* test. Because the six T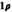 ratios investigated were not independent (each FSL was used in three comparisons), the Benjamini–Hochberg correction was not valid; thus, the Bonferroni correction [56] was used to account for multiple comparisons (n=6), resulting in a significance threshold of p<0.0083.

## 3 Results

All mice from Groups 2 and 3 developed tumours with the exception of one mouse from Group 3. Most of the tumours were MRI visible by Week 3; they grew rapidly and all tumour‐bearing mice were terminated on Week 3 or 4.

**FIGURE 3:**
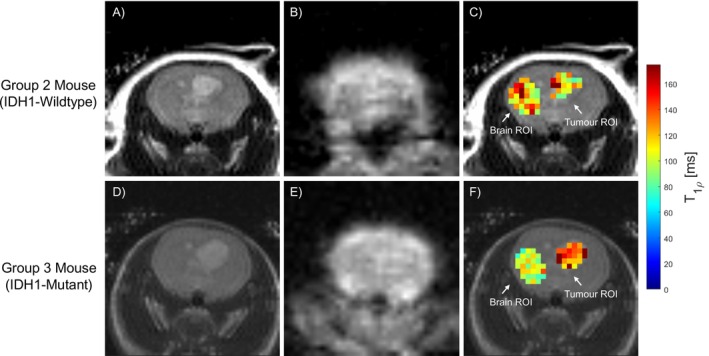
Same‐slice axial images of a representative mouse from Group 2 (IDH1 wild type; top row, Panels A–C) and Group 3 (IDH1 mutant; bottom row, Panels D–F), taken at Week 3. (A,D) T
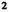
‐FSE anatomical image, where the tumour can be seen as the lighter contrast within the brain. (B,E) T
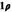
‐weighted image acquired with TSL = 15 ms and FSL = 2000 Hz. (C,F) T
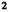
‐FSE with T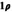 map from FSL = 2000 Hz overlaid within the ROIs for brain and tumour.

Example T
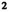
‐FSE and T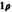 images from representative Groups 2 and 3 mice are displayed in Figure 3, along with T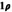 maps of brain and tumour ROIs. It can be seen from the T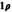 maps (Panels C and F) that the Group 2 (IDH1 wild type) mouse exhibited similar T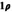 values between the brain and tumour ROIs, while the Group 3 (IDH1 mutant) mouse presented higher T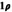 values in the tumour compared with the brain ROI. This was a trend seen across most tumour‐bearing mice in each group.

**FIGURE 4:**
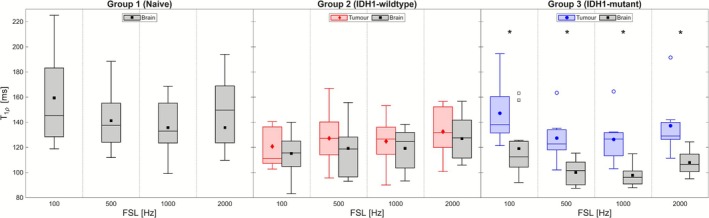
Comparisons of T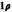 values in brain and tumour ROIs for the three groups at each FSL. Boxes depict sample median along with upper and lower quartiles, with whiskers denoting the maximum and minimum values of the sample. Group means are plotted as filled markers, while open markers denote outliers (points more than 1.5 times the interquartile range away from upper/lower quartiles). Statistically significant differences in ROI values within an FSL are highlighted by *.

Brain and tumour T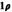 values are plotted for all three groups at each FSL in Figure 4; averages for each group are recorded in Table 2. Results from the statistical tests comparing T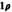 values between groups and ROIs are tabulated in Table 3 under the ‘T
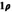
’ header. It was found that for Group 2, T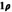 values in the tumour were not significantly different than those in the brain at any FSL; however, Group 3 exhibited significantly higher tumour T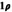 values compared with the brain at all FSL. A direct comparison of tumour T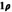 values between Groups 2 and 3 found no significant differences at any FSL. Brain T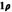 values were found to be significantly different at all FSLs between Groups 1 and 2, as well as between Groups 1 and 3, and brain T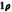 values were found to be significantly different between Groups 2 and 3 at all FSL except 100 Hz.

**TABLE 2:** Mean (± standard deviation) T metrics for Group 1 (naïve), Group 2 (IDH1 wild type), and Group 3 (IDH1 mutant), at each FSL. T values of brain and tumour ROIs for each group are listed, as well as ΔT values for Groups 2 and 3.

	Average T 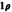 (ms)					Average ΔT 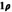 (%)	
	Group 1	Group 2		Group 3		Group 2	Group 3
FSL (Hz)	Brain	Brain	Tumour	Brain	Tumour		
100	159±36	115±16	121±16	119±22	147±25	2±13	27±18
500	141±22	119±21	127±21	100±10	127±19	6±13	31±15
1000	136±20	119±15	125±18	98±9	126±20	3±11	29±13
2000	148±26	127±16	133±19	108±10	137±26	2±14	28±15

**TABLE 3:** Results of the Mann–Whitney *U* tests comparing T metrics (T and ΔT) between Group 1 (G1; naïve), Group 2 (G2; IDH1 wild type), and Group 3 (G3; IDH1 mutant). p values are listed for each comparison at each FSL; statistically significant results following Benjamini–Hochberg correction with FDR = 5% are denoted by *.

	T 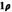						ΔT 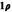
	Tumour vs. brain		Tumour	Brain			
FSL (Hz)	G2	G3	G2 vs. G3	G1 vs. G2	G1 vs. G3	G2 vs. G3	G2 vs. G3
100	0.6366	0.0130*	0.0229	0.0003*	0.0019*	0.9795	0.0115*
500	0.4310	0.0008*	0.9182	0.0174*	< 0.0001*	0.0109*	0.0052*
1000	0.5923	0.0008*	0.9182	0.0424*	< 0.0001*	0.0009*	0.0012*
2000	0.5495	0.0003*	0.9182	0.0343*	0.0001*	0.0031*	0.0052*

**FIGURE 5:**
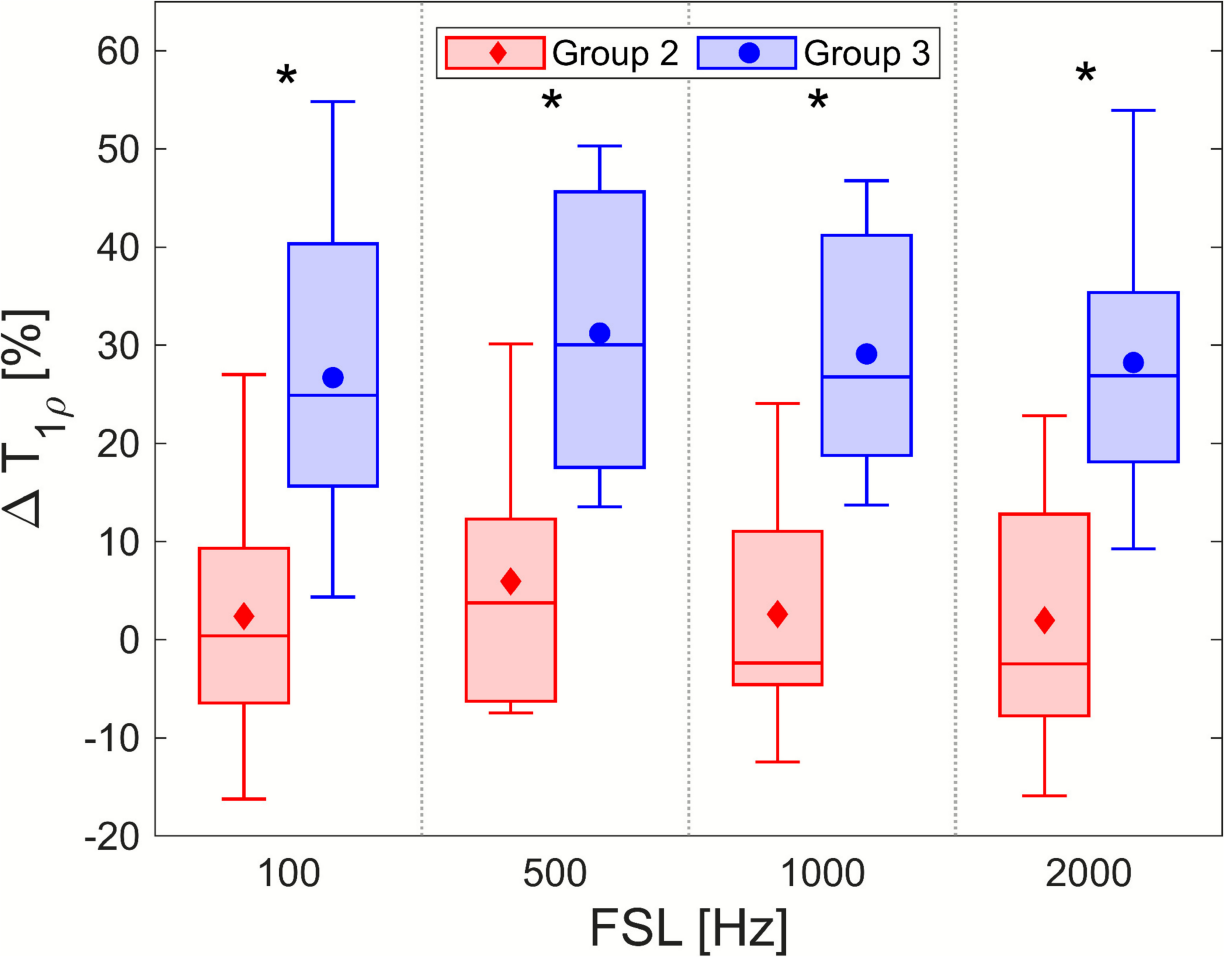
Comparisons of ΔT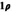 values between Group 2 (IDH1 wild type) and Group 3 (IDH1 mutant) mice at each FSL. Boxes depict sample median along with upper and lower quartiles, with whiskers denoting the maximum and minimum values of the sample. Group means are plotted as filled markers. Statistically significant differences in group values within an FSL are highlighted by *.

Figure 5 plots the ΔT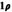 values for Groups 2 and 3 mice at each FSL; average values for the groups are listed in Table 2, and results of the statistical tests comparing them are recorded in Table 3 under the 'ΔT
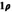
' header. ΔT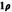 was found to be significantly different between Groups 2 and 3 at each FSL: Group 2 exhibited little difference between brain and tumour T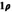 values, while Group 3 tumour T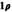 values were elevated compared with the brain.

**FIGURE 6:**
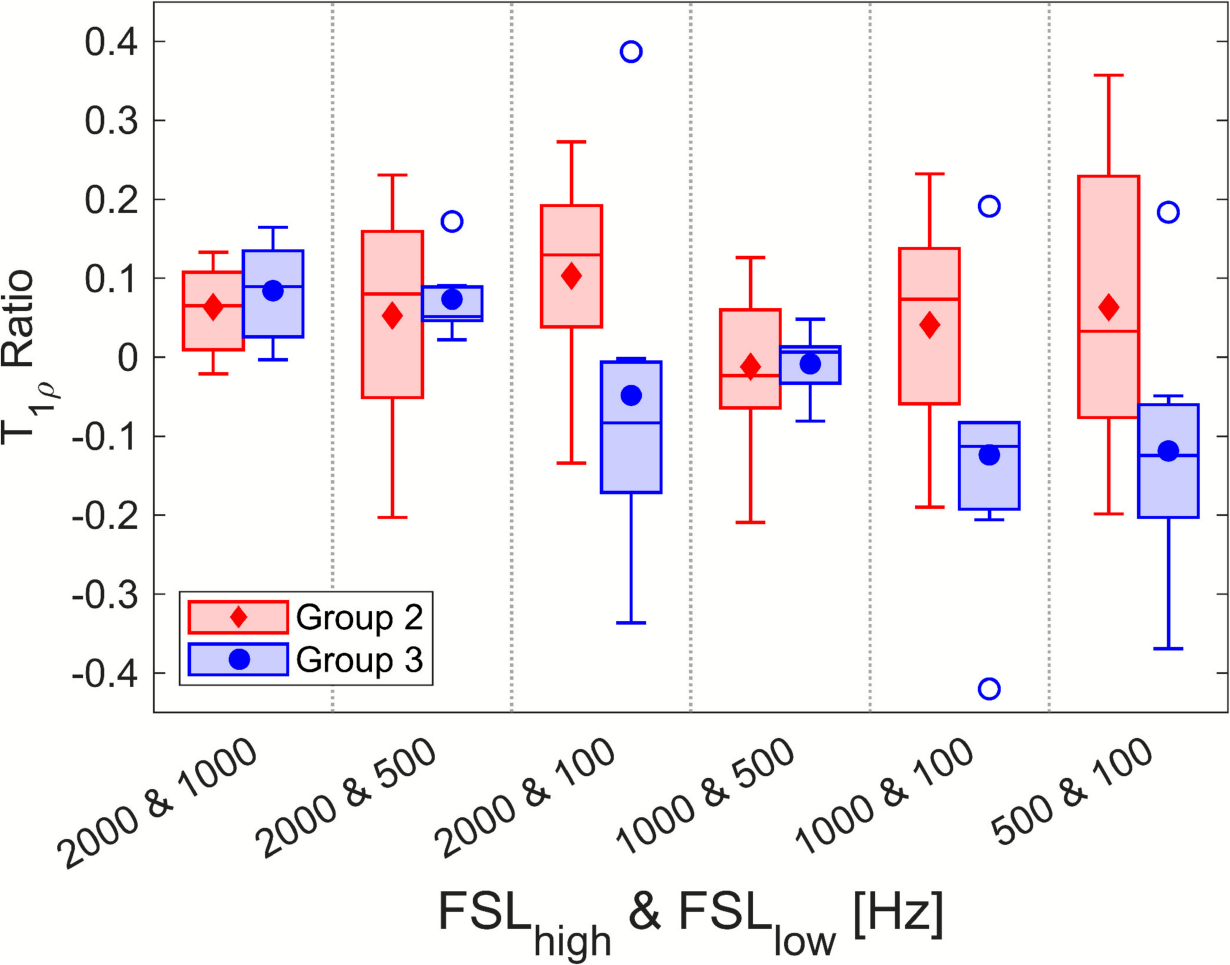
Comparisons of normalised T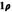 dispersion ratios in tumour ROIs between Groups 2 and 3 at each FSL combination. Boxes depict sample median along with upper and lower quartiles, with whiskers denoting the maximum and minimum values of the sample. Group means are plotted as filled markers, while open markers denote outliers (points more than 1.5 times the interquartile range away from upper/lower quartiles).

The T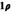 dispersion ratios are plotted in Figure 6 for Groups 2 and 3 tumour ROIs; results of the statistical tests comparing these metrics between the two groups are tabulated in Table 4. Tumour T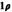 dispersion ratios were not found to be significantly different between the two groups at any of the six FSL combinations.

**TABLE 4:** Results of the Mann–Whitney *U* tests comparing tumour T dispersion ratios between Groups 2 and 3; p values are listed for each combination of FSL and FSL. No significant differences were found between Groups 2 and 3 at any FSL combination.

	FSL 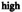 (Hz)		
FSL 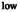 (Hz)	2000	1000	500
1000	0.6065	—	—
500	0.9182	0.9313	—
100	0.1416	0.0907	0.0712

**FIGURE 7:**
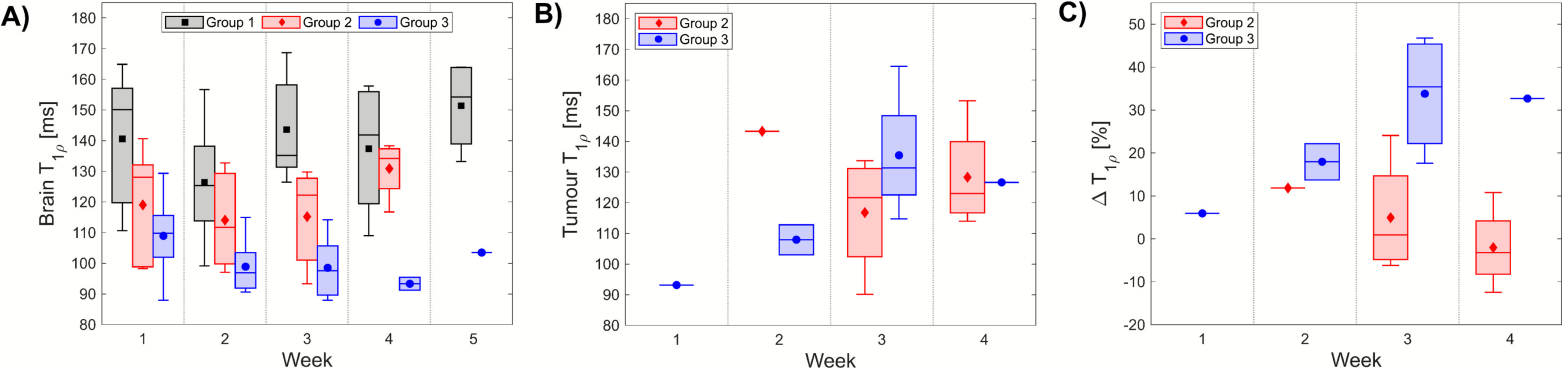
T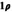 metrics at FSL = 1000 Hz plotted week‐wise. Boxes depict sample median along with upper and lower quartiles, with whiskers denoting the maximum and minimum values of the sample. Group means are plotted as filled markers. (A) Brain T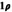 values for each group. (B) Tumour T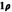 values for tumour‐bearing mice. (C) ΔT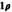 values for tumour‐bearing mice.

Although there were no significant changes in T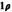 of tumour or brain tissue between any individual weeks, these values did exhibit trends over the course of the study. To further explore any temporal behaviour during tumour development, T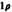 values were plotted week‐wise for mice in each group: Figure 7A displays brain values, and Figure 7B displays tumour values, both at FSL = 1000 Hz. Figure 7A depicts no visible changes in brain T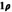 over time for Group 1 and no large changes in brain T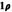 over time for Group 2; however, there appear to be larger drops in brain T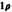 of Group 3 over time. Figure 7B shows tumour T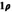 values generally increase over time for Group 3, while those of Group 2 exhibit a less clear temporal trend. The compounded effect of brain and tumour behaviour over time is captured by ΔT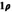, which is plotted week‐wise in Figure 7C for FSL = 1000 Hz. Groups 2 and 3 exhibit clear and opposite trends over the course of the study: Group 2 sees a decrease in ΔT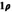 over time, while Group 3 sees an increase. These trends become more apparent with higher FSL and are most pronounced at FSL = [1000, 2000] Hz.

## 4 Discussion

Our findings show that IDH1‐wild‐type tumours exhibit little difference in T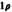 values from the brain, while IDH1‐mutant tumours present significantly higher tumour T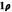 values compared with the brain. This was found to not only be a feature of the tumour values themselves but arising from differences in brain T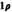 values. A direct comparison of IDH1‐mutant and ‐wild‐type tumour T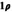 values found no significant differences between them, but we did, however, find that tumour‐bearing mice had significantly different brain T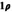 values than their naïve counterparts, and brain T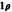 values even differed between IDH1‐mutant and ‐wild‐type groups at high FSL.

It is possible, however, that these T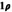 differences could be driven by differences of group T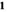 and T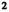 values used to correct the T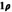 values (Table 1). Uncorrected T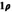 metrics and statistical analyses have been included in the Supporting Information; no significant differences in T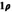 of brain ROIs were found in the uncorrected data (Table S2). Yet, limited clinical studies have demonstrated sensitivity of both T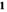 [57] and T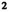 [57, 58, 59] relaxation times to IDH mutation status in glioma. Both have been reported to be significantly higher in IDH‐mutant glioma compared with wild type, as reflected by our measurements as well. Further characterisation of relaxometry and IDH sensitivity would be beneficial, especially regarding the value of T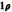 in a multiparametric analysis.

Nevertheless, the difference of brain T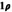 values between the naïve, IDH1‐wild‐type and IDH1‐mutant groups may demonstrate the effect of glioma on the whole brain. It is known that the influence of glioma extends beyond the tumour borders—its presence can cause changes across the entire brain including inflammation and immune cell response [60], compromised blood brain barrier integrity [61], and disrupted neural activity [62]. Being a diffuse tumour, it is especially infiltrative, with cells spreading throughout the brain without inducing morphological changes [63]. T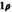, being sensitive to the molecular environment and processes in the extracellular matrix, may thus provide insight into the whole‐brain response to glioma.

The metric ΔT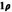, which normalises tumour T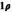 to healthy brain tissue, captures the distinction between IDH1‐mutant and ‐wild‐type glioma cases. We found ΔT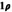 to be significantly different between IDH1‐mutant and ‐wild‐type tumours: While IDH1‐wild‐type tumours have T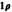 values similar to those of brain (∼3% difference on average), IDH1‐mutant tumours see elevated T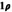 values compared with brain (∼29% difference on average).

This is not the first study to record similar T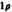 values between IDH‐wild‐type glioma and brain; preclinical studies using a rat model with BT4C glioma (which is IDH wild type [64]) have reported very little contrast between tumour and brain prior to treatment [34, 43, 44]. While, to our knowledge, no preclinical studies of IDH‐mutant glioma have been performed with T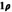, a small number of clinical studies have evaluated the IDH sensitivity of T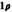 [39, 40, 41]. Very recently, Dai et al. [41] have reported significantly higher relative T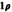 in IDH‐mutant glioma compared with IDH‐wild‐type glioma (where relative T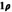 = tumour T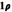/brain T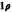) at FSL = [100, 200, 500] Hz. Their result is in agreement with our own; the normalised difference between glioma and brain T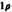 values (e.g., ΔT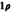) exhibits strong potential as an IDH1 biomarker. As well described by Dai et al. [41], shorter T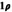 times in IDH‐wild‐type glioma can be attributed to a denser extracellular matrix and increased macromolecular components, thus restricting the motion of water molecules and promoting more chemical exchange between water and macromolecules, which accelerates the T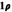 relaxation process, shortening the T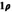 time.

The T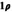 dispersion ratios explored did not yield any significant distinction between IDH1‐mutant and ‐wild‐type tumours. Like Kettunen et al. [43], we found that making T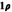 measurements at multiple FSL did not offer anything beyond T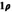 at a single FSL. T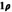 dispersion remains poorly studied and understood; future investigations with larger sample sizes may yet reveal its utility. It is noted, however, that ΔT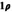 differences between IDH1‐mutant and ‐wild‐type groups were the most apparent at FSL = 1000 Hz, and T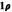 could only distinguish between brains of IDH1‐mutant and ‐wild‐type groups at FSL ≥500 Hz. Similarly, Sierra et al. [44] noted higher sensitivity of T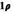 to treatment response with higher FSL (over the range of ∼85–6000 Hz). Future studies may thus benefit from implementing high FSL on the order of kHz if technically feasible.

The week‐wise breakdown of brain T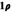 measurements (Figure 7A) suggests that for IDH1‐mutant mice, T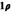 values in the brain generally decrease as the tumour progresses—potentially even before tumours are visible, such as at Week 2. Meanwhile, IDH1‐wild‐type mice see little change in brain T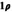 values over time until perhaps Week 4, at which point they may see an increase. The tumour T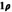 values for the two groups over time (Figure 7B) implies that tumour T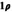 values in IDH1‐mutant mice increase with time, while the trend of tumour values for IDH1‐wild‐type mice is less clear. However, the week‐wise breakdown of ΔT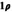 (Figure 7C) depicts opposite trends between IDH1‐mutant and ‐wild‐type mice over the course of tumour development. Though the sample sizes for these groups are especially small at some weeks, ΔT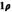 values appear to increase over time in IDH1‐mutant mice and decrease in IDH1‐wild‐type mice. This suggests that ΔT
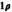
—which is sensitive to changes in both the tumour and the ‘healthy’ brain—may capture differences in the invasiveness of and immune response to the different tumour types, and may be indicative of early tumour effects. However, because the T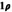 corrections were performed with T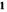 and T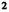 values acquired near the end of the study (Week 4), it is not clear to what extent the temporal trends observed in T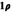 and ΔT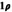 are driven by temporal changes in T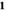 and T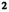 that have been overlooked. Yet, other studies have demonstrated T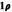 to be sensitive to treatment‐induced changes in glioma as early as 3–4 days after starting treatment [34, 43, 44], and the temporal trends seen in ΔT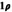 are present in the uncorrected data as well (Figure S3C). ΔT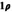 thus suggests potential to serve as a prognostic indicator and marker of therapeutic efficacy and may even reveal more than T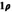 alone. A more thorough exploration of this trend evaluated temporally with a larger sample size may help us to better understand the brain's response to glioma and IDH1 typing.

This study had a number of limitations: (1) The sample size was small, with only n=5 mice per group. T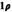 measurements were collapsed across Weeks 2–4 to permit larger n for statistical robustness; however, this could induce bias from overrepresentation of mice who survived more than one week with a tumour and may overlook time‐dependent changes in the tumour and/or brain (as Figure 7 suggests). Further investigation with larger sample sizes will aid in discerning the diagnostic utility of T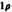 with respect to IDH1 typing in glioma and may reveal additional insight of the sensitivity of T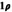 to time‐dependent changes. (2) Due to scan time considerations, we could not acquire T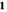 and T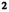 maps in each mouse at every week. Thus, T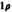 multislice correction was performed with group average T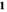 and T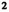 values, rather than individual maps at each time point. It is possible that this approximation overlooked time dependence and/or individual variation in T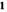 and T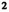, affecting corrected T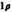 times. T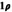 metrics and statistical analyses prior to correction have been included in the Supporting Information; while the uncorrected data do exhibit different results from the corrected data with respect to the T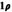 value comparisons, the findings of ΔT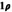 remain consistent to a high degree of statistical significance irrespective of correction. Nevertheless, it would behove future studies to collect T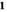 and T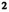 maps in each scan session. (3) T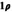 maps were low resolution due to limitations of the EPI sequence, which could affect the robustness of the measurements. Higher resolution T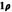 maps may also permit analysis of different tumour regions, as has been explored clinically [39]. It is possible that differences in T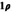 metrics between Groups 2 and 3 could be attributed to tumour heterogeneity (e.g., oedema and necrosis); however, a visual examination of the anatomical images did not yield obvious differences in tumour heterogeneities between groups. (4) T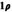 values exhibited some variability between mice and across weeks. This is one of the reasons that ΔT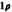 was used: to normalise tumour values to the brain of each mouse and therefore minimise the impact of individual variation. While future studies amending these constraints may better characterise the diagnostic utility of T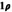 for IDH1 typing in glioma, this preliminary investigation lends promise to its efficacy.

## 5 Conclusions

To our knowledge, this is the first preclinical study investigating T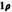 as a biomarker for IDH1 mutation status in glioma. We found that a measurement of tumour T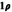 normalised to the brain (ΔT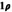) was capable of distinguishing IDH1‐mutant and ‐wild‐type glioma across a wide range of spin‐lock frequencies. IDH1‐wild‐type glioma exhibits little difference in tumour and brain T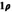 values (ΔT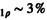), while IDH1‐mutant tumours present significantly higher T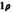 values in tumour than brain (ΔT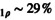). Though further investigations with larger sample sizes are required to better characterise and understand these findings, ΔT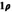 demonstrates potential to serve as a non‐invasive biomarker for IDH1 mutation status in glioma.

## Author Contributions

**H.J.S.E.:** T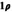 pulse sequence creation, data acquisition, data processing and analysis, manuscript writing. **S.S.:** cell culturing, animal procedures, manuscript review. **C.D.:** animal procedures. **K.B.:** study conception and design, project oversight, animal procedures, manuscript review. **J.R.:** study conception and design, project oversight, manuscript review.

## Conflicts of Interest

The authors declare no conflicts of interest.

## Supporting information



supplementary.pdf.

## Data Availability

The data that support the findings of this study are available from the corresponding author upon reasonable request.
