# Supplementary material for: T as a Biomarker for IDH1 Mutation Status in a Glioma Mouse Model
Source: NMR Biomed. 2025 Dec 23;39(2):e70214. doi: 10.1002/nbm.70214 (PMC12727679; doi:10.1002/nbm.70214)
Supplement: Supplementary file 1 — supplementary.pdf. [file NBM-39-e70214-s001.pdf]

Supplementary Material:

$T_{1\rho}$ as a biomarker for IDH1 mutation status in a glioma mouse model

Hannah J.S. Ehler, Saki Sultana, Christa Davis, Kimberly Brewer, James Rioux

	Average $T_{1\rho}$ [ms]					Average $\Delta T_{1\rho}$ [%]	
	Group 1	Group 2		Group 3		Group 2	Group 3
FSL [Hz]	Brain	Brain	Tumour	Brain	Tumour		
100	70 ± 7	65 ± 5	71 ± 6	72 ± 8	86 ± 9	7 ± 7	21 ± 9
500	68 ± 5	67 ± 7	74 ± 6	65 ± 4	79 ± 7	9 ± 7	24 ± 8
1000	66 ± 6	67 ± 6	74 ± 6	64 ± 4	78 ± 8	8 ± 7	22 ± 7
2000	68 ± 6	69 ± 4	75 ± 6	68 ± 4	82 ± 9	8 ± 8	22 ± 8

Table S1: Mean ($\pm$ standard deviation) **uncorrected** $T_{1\rho}$ metrics for group 1 (naïve), group 2 (IDH1-wildtype), and group 3 (IDH1-mutant), at each FSL. $T_{1\rho}$ values of brain and tumour ROIs for each group are listed, as well as $\Delta T_{1\rho}$ values for groups 2 and 3.

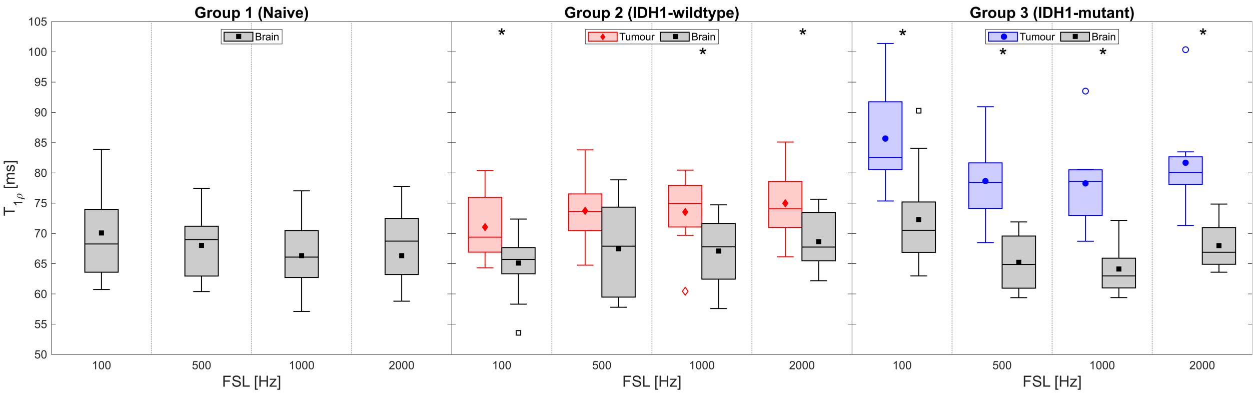

Figure S1: Comparisons of **uncorrected** $T_{1\rho}$ values in brain and tumour ROIs for the three groups at each FSL. Boxes depict sample median along with upper and lower quartiles, with whiskers denoting the maximum and minimum values of the sample. Group means are plotted as filled markers, while open markers denote outliers (points more than 1.5 times the interquartile range away from upper/lower quartiles). Statistically significant differences in ROI values within an FSL are highlighted by *.

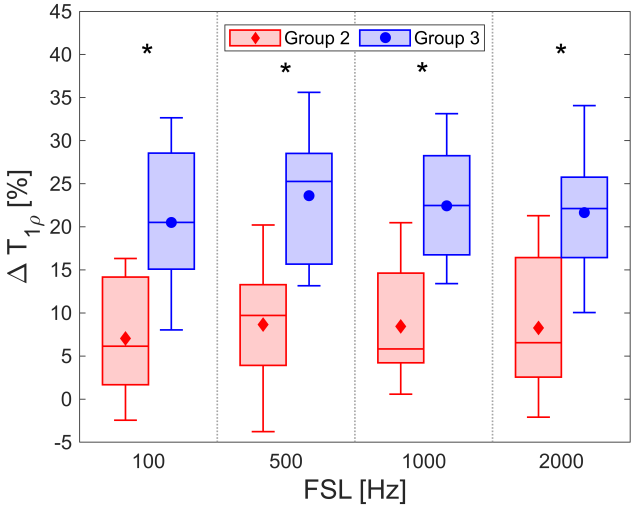

Figure S2: Comparisons of **uncorrected** $\Delta T_{1\rho}$ values between group 2 (IDH1-wildtype) and group 3 (IDH1-mutant) mice at each FSL. Boxes depict sample median along with upper and lower quartiles, with whiskers denoting the maximum and minimum values of the sample. Group means are plotted as filled markers. Statistically significant differences in group values within an FSL are highlighted by *.

	$T_{1\rho}$						$\Delta T_{1\rho}$
	Tumour vs Brain		Tumour	Brain			
FSL [Hz]	G2	G3	G2 vs G3	G1 vs G2	G1 vs G3	G2 vs G3	G2 vs G3
100	0.0253 *	0.0072 *	0.0021 *	0.0576	0.5101	0.0193	0.0033 *
500	0.0547	0.0005 *	0.2105	0.6784	0.1796	0.6620	0.0021 *
1000	0.0107 *	0.0002 *	0.3510	0.6784	0.2515	0.1729	0.0033 *
2000	0.0153 *	0.0003 *	0.1142	0.7767	1	0.6620	0.0079 *

Table S2: Results of the Mann-Whitney U-tests comparing **uncorrected** $T_{1\rho}$ metrics ($T_{1\rho}$ and $\Delta T_{1\rho}$) between group 1 (G1; naïve), group 2 (G2; IDH1-wildtype), and group 3 (G3; IDH1-mutant). p-values are listed for each comparison at each FSL; statistically significant results following Benjamini-Hochberg correction with FDR = 5% are denoted by *.

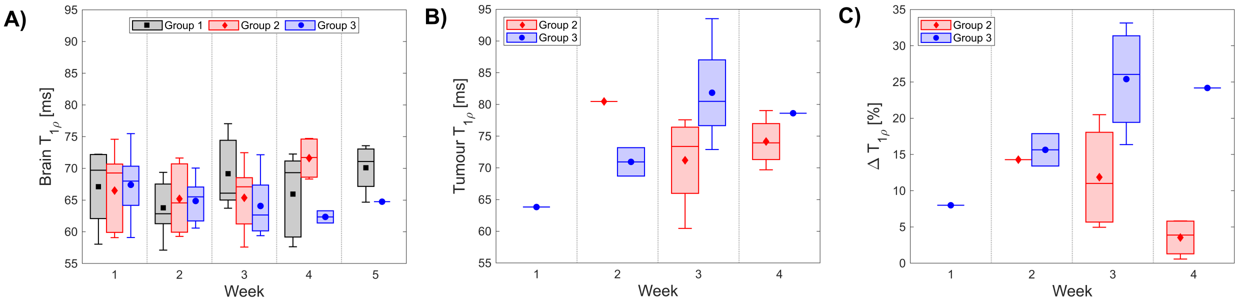

Figure S3: **Uncorrected** $T_{1\rho}$ metrics at FSL = 1000 Hz plotted week-wise. Boxes depict sample median along with upper and lower quartiles, with whiskers denoting the maximum and minimum values of the sample. Group means are plotted as filled markers. **A)** Brain $T_{1\rho}$ values for each group. **B)** Tumour $T_{1\rho}$ values for tumour-bearing mice. **C)** $\Delta T_{1\rho}$ values for tumour-bearing mice.
